# CamelinaWeed: an expert-agronomist-annotated UAV RGB and multispectral dataset for weed and crop monitoring in *Camelina sativa*

**DOI:** 10.1016/j.dib.2026.113135

**Published:** 2026-08-05

**Authors:** Ioannis N. Rallis, Kyriakos Kempapidis, Panagiotis Raptis, Emmanuel K. Raptis, Athanasios Ch. Kapoutsis

**Affiliations:** a Department of Electrical and Computer Engineering, Democritus University of Thrace, Xanthi, Greece; b BIOS Agrosystems SA, Sindos Industrial Area, Thessaloniki, Greece; c Information Technologies Institute, Centre for Research and Technology Hellas (CERTH), Thessaloniki, Greece; d Athena Research Center, Institute for Language and Speech Processing, Xanthi, Greece

**Keywords:** Precision agriculture, Weed mapping, Remote sensing, Computer vision, Semantic segmentation

## Abstract

This dataset provides UAV-based RGB and multispectral imagery for crop monitoring, weed mapping, and field-level analysis in *Camelina sativa* cultivation. Data were collected from three agricultural fields in Thessaloniki and Chalkidiki, Greece, during summer 2025 and winter 2025–2026, capturing variability across locations, seasons, crop growth stages, UAV platforms, flight altitudes, spatial resolutions, illumination conditions, and sensing modalities. The dataset includes 3023 manually annotated RGB UAV images with human expert-generated polygon annotations of weed instances. The annotation scheme includes both coarse weed categories, such as broadleaf, narrowleaf, and generic weed classes, and fine-grained species-level labels, supporting classification, object detection, semantic and instance segmentation, hierarchical learning, and weed distribution analysis. In addition, the dataset provides RGB and multispectral UAV imagery, the raw RGB and multispectral images used for orthomosaic reconstruction, and both RGB and multispectral orthomosaic products in GeoTIFF format. The data were acquired using DJI Phantom 4 Pro and DJI Mavic 3 M UAV platforms at different flight altitudes, resulting in multiple ground sampling distances and image resolutions. This dataset is intended to support the development, benchmarking, and validation of computer vision and precision agriculture methods under realistic field conditions. To the best of our knowledge, it is among the first publicly available UAV datasets specifically focused on weed monitoring and field analysis in *Camelina sativa* crops.

Specifications Table

**Table:** 

Subject	Computer Sciences
Specific subject area	UAV-based remote sensing and precision agriculture in *Camelina sativa* fields
Type of data	Annotated and Raw RGB images (JPEG) Raw multispectral images (TIFF) Orthomosaics (GeoTIFF) Polygon annotations (JSON)
Data collection	Data were collected using a DJI Phantom 4 Pro UAV equipped with an RGB camera and a DJI Mavic 3 M equipped with RGB and multispectral cameras. Images were collected during UAV coverage missions *over Camelina sativa (*L.*) Crantz* fields, with the camera gimbal adjusted to −89° vertically to the field. Image acquisition was performed at flight altitudes of 2 m, 3 m, 5 m, and 10 m, depending on the field and UAV platform. The dataset includes data collected from three agricultural fields in Thessaloniki and Chalkidiki, Greece, during summer and winter cultivation periods.
Data source location	Winter cultivation field (Thessaloniki, Greece): [40.766551, 22.993202; 40.766327, 22.994137; 40.767043, 22.994564; 40.767380, 22.993470] Winter cultivation field (Chalkidiki, Greece): [40.368424, 23.068174; 40.368555, 23.069137; 40.369965, 23.068723; 40.369791, 23.067736] Summer cultivation field (Thessaloniki, Greece): [40.565772, 22.990067; 40.566154, 22.991342; 40.568324, 22.989971; 40.567646, 22.988174; 40.566623, 22.988909]
Data accessibility	Repository name: Zenodo Data identification number: https://doi.org/10.5281/zenodo.20148697 Direct URL to data: https://zenodo.org/records/20148697 Instructions for accessing these data: The complete dataset is publicly available through the Zenodo repository. Dataset documentation, annotation details, directory structure, and visualization tools are additionally provided through the public GitHub repository: https://github.com/aura-laboratory/CamelinaWeed-Annotated-UAV-Imagery-Dataset-for-Camelina-sativa
Related research article	None

## 1 Value of the Data

• The dataset fills a gap in the literature by providing UAV imagery from *Camelina sativa* fields, offering examples for training and testing automated weed detection models in this underrepresented crop.
• The dataset was collected under real field conditions using UAV platforms, includes EXIF metadata, and was acquired at different flight altitudes, supporting precision agriculture applications, simulation studies for autonomous agricultural robots, and the evaluation of scale variation across spatial resolutions.
• Data acquired from multiple fields in Chalkidiki and Thessaloniki and across different seasons (summer and winter) enable the study of model generalization under varying environmental conditions, crop growth stages, and illumination, as well as potential variations in weed distribution.
• The annotations include both generic categories (e.g., broadleaf, narrowleaf, weed) and more detailed subclasses, enabling flexible use of the dataset for different levels of analysis.
• The dataset supports classification, detection, segmentation, and hierarchical learning tasks, while also enabling downstream applications such as automated weeding, crop monitoring, yield protection, and decision-support systems for more sustainable agricultural management.

## 2 Background

*Camelina sativa* is an emerging oilseed crop of increasing interest in sustainable agriculture and is recognized in North America and Europe as a low-input crop with valuable applications [[1], [2], [3]]. However, weed management remains a major challenge for its successful commercial production [4], highlighting the need for effective weed monitoring during cultivation. The dataset was compiled to provide UAV-based RGB and multispectral imagery from *Camelina sativa* fields under real agricultural conditions. Data were collected from fields in Thessaloniki and Chalkidiki, Greece, during summer and winter cultivation periods, using different UAV platforms, flight altitudes, spatial resolutions, and sensing modalities. The dataset documents crop and weed variability at both image and field scale, including annotated RGB images for weed detection and segmentation, raw RGB and MS (multispectral) imagery, and orthomosaic products for broader field-level analysis.

As summarized in Table 1, existing public UAV crop–weed datasets primarily focus on cotton, sugar beet, and maize and generally provide either broad crop–weed labels, a limited number of weed species, or restricted variability in acquisition conditions. At the same time, recent multi-scale and context-aware aerial-image studies [5,6] emphasize the importance of data that capture variations in object scale, local detail, and broader scene context. To the best of our knowledge, CamelinaWeed is among the first public UAV datasets specifically developed for weed monitoring in *Camelina sativa*. It addresses this research gap by combining expert polygon annotations, hierarchical broadleaf, narrowleaf, and species-level labels, naturally imbalanced weed distributions, weed-positive and weed-negative images, and RGB and multispectral imagery acquired across multiple fields, seasons, UAV platforms, flight altitudes, and spatial resolutions. These characteristics provide a dedicated benchmark for evaluating weed detection, segmentation, hierarchical learning, and multi-scale or context-aware methods under realistic agricultural conditions.

**Table 1:** Compact comparison of representative publicly available UAV crop-weed datasets.

Dataset	Crop / sensor	Dataset size	Acquisition diversity
CoFly-WeedDB [7]	Cotton / RGB	201 images	One location; one campaign
WeedMap [8]	Sugar beet / MS	8 orthomosaics	Two locations; two campaigns
PhenoBench [9]	Sugar beet / RGB	2872 images	Multiple acquisition dates
WeedsGalore [10]	Maize / RGB–MS	156 tiles; 4 orthomosaics	Four acquisition dates
**CamelinaWeed** [11]	***Camelina sativa* / RGB–MS**	**3023 annotated images**	**3 fields; 2 seasons; 2 UAVs; multiple altitudes and GSDs**

## 3 Data Description

The presented dataset captures weed occurrences in *Camelina sativa* crops across three agricultural fields in Chalkidiki and Thessaloniki, Greece. It was compiled to provide a targeted UAV image dataset for *Camelina sativa* cultivation, supporting the development of automated weed detection approaches and providing a practical resource for field management and crop monitoring. Data were collected during two distinct time periods, namely summer 2025 and winter 2025–2026, corresponding to different cultivation stages and growing conditions, with the *Camelina sativa* crop maintaining healthy growth conditions throughout the acquisition period.

The dataset includes annotated RGB UAV images in JPEG format, polygon-based annotation files in JSON format, raw RGB and multispectral imagery, and orthomosaic products in GeoTIFF format. The annotations include both generic weed categories and species-level labels, supporting weed detection, classification, and segmentation tasks. The acquired UAV data are summarized in Table 2, Table 3.

**Table 2:** Summary of the acquired UAV data for orthomosaic generation.

Season	Location	Acquisition setting	Images	Orthomosaic
Winter 2025–2026	Thessaloniki	Mavic 3 M flight at 20 m altitude RGB	227	✔
		Mavic 3 M flight at 20 m altitude MS	908	✔
Winter 2025–2026	Chalkidiki	Mavic 3 M flight at 20 m altitude RGB	1351	✔
		Mavic 3 M flight at 20 m altitude MS	5404	✔

**Table 3:** Summary of the acquired UAV data for weed detection.

Season	Location	Acquisition setting	Weed-positive images	Weed-negative images
Summer 2025	Thessaloniki	Phantom flight at 5 m altitude	34	32
		Phantom flight at 10 m altitude	297	46
Winter 2025–2026	Thessaloniki	Phantom flight at 3 m altitude	17	32
		Mavic 3 M flight 1 at 2 m altitude	627	215
		Mavic 3 M flight 1 at 2 m altitude MS	−	842
		Mavic 3 M flight 2 at 2 m altitude	47	193
		Mavic 3 M flight 2 at 2 m altitude MS	−	240
Winter 2025–2026	Chalkidiki	Phantom flight at 3 m altitude	43	159
		Phantom flight at 5 m altitude	55	144

Table 2 provides an overview of the UAV data that can be used for orthomosaic generation tasks. These data include both RGB and multispectral imagery together with the corresponding orthomosaic products generated from the 20 m flights. The raw images in this table were used for orthomosaic creation and were not annotated for weed detection. Therefore, they provide complementary spatial information for large-scale field analysis and visualization of weed distribution patterns across the examined agricultural areas.

**Fig. 1:**
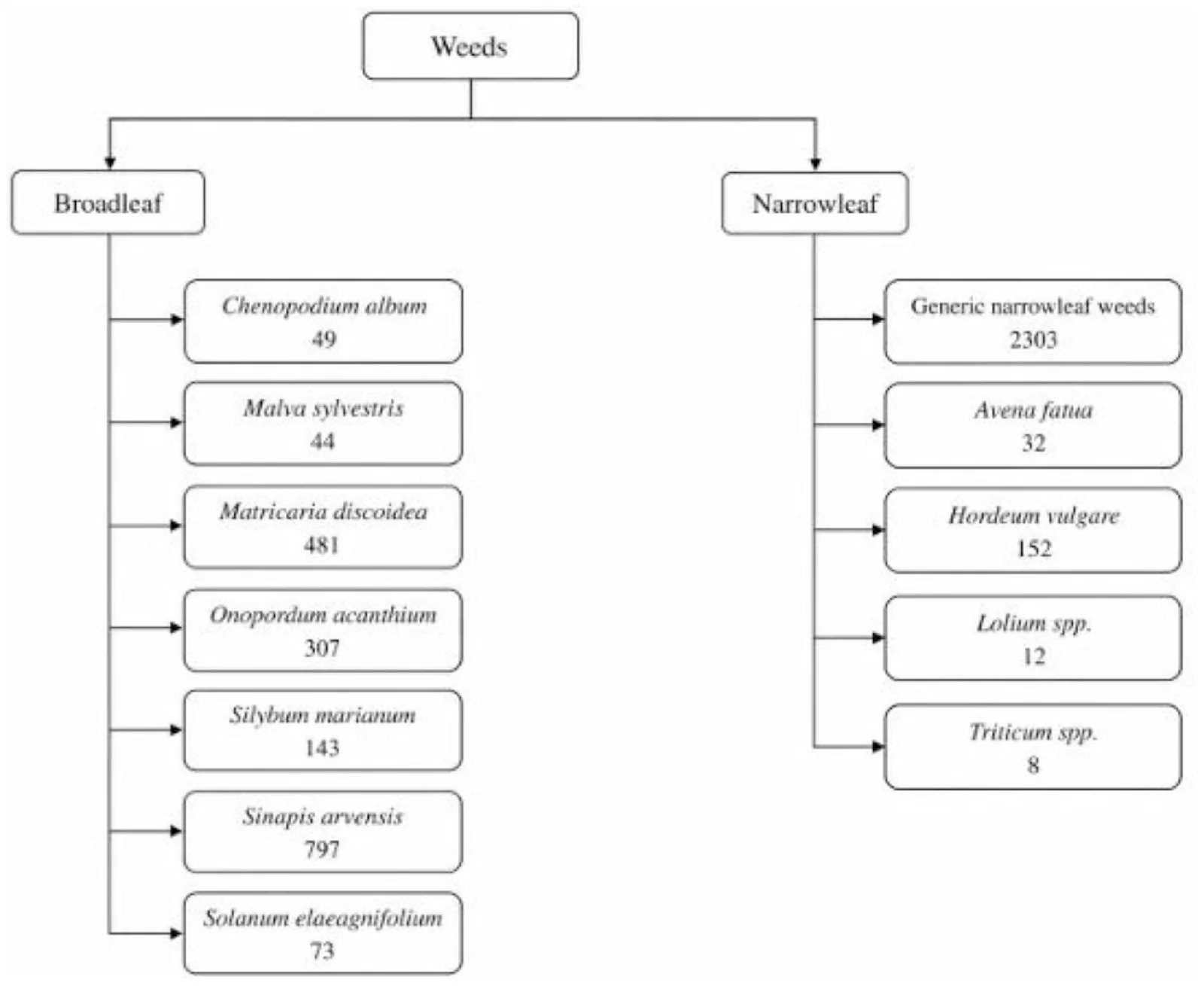
Hierarchical organization of weed instances in the dataset. Weed samples are grouped into Broadleaf and Narrowleaf categories, with each terminal node representing a weed species or generic weed group together with the corresponding number of instances.

Table 3 summarizes the UAV data that can be used for weed detection tasks. The dataset contains 3023 UAV images, including 1120 images with visible weed presence and 1903 images where no weeds were identified. Weed instances were manually annotated by expert agronomists using polygon-based segmentation in the Roboflow platform [12]. The annotations include both species-level labels and broader categories, such as broadleaf and narrowleaf weeds, as illustrated in Fig. 1. This hierarchical annotation structure enables analysis at different levels of detail, ranging from general weed classification to fine-grained species recognition.

All images in the dataset were acquired using different UAV platforms, resulting in varying spatial resolutions. Images captured with the DJI Phantom 4 Pro have a resolution of 5472 × 3078 pixels, while images acquired with the DJI Mavic 3 M have a resolution of 1920 × 1080 pixels. This variability enables the evaluation of models under different spatial scales, acquisition settings, and sensing modalities.

The dataset includes annotated weed instances organized into two main categories, as shown in Fig. 1: broadleaf and narrowleaf weeds. The broadleaf group contains seven species-level classes, while the narrowleaf group includes one generic narrowleaf class and four species-level classes. This hierarchical organization enables experiments at different levels of classification granularity. The number of annotated instances varies across categories and species, reflecting the natural occurrence of weeds in the examined fields. This class imbalance should therefore be considered during model training and evaluation.

**Fig. 2:**
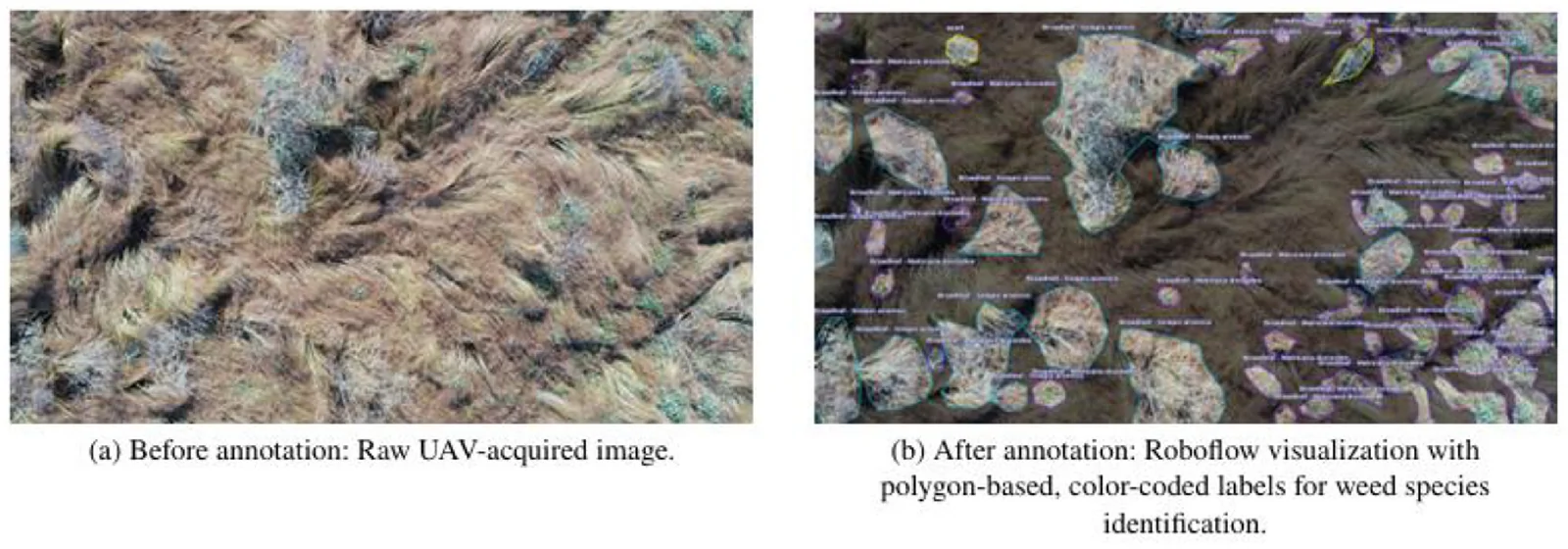
Example of the annotation format used in the dataset within Roboflow. The first image presents the raw UAV-acquired image, while the second image shows the polygon-based annotations visualized in the Roboflow environment, with the corresponding class labels displayed.

As illustrated in Fig. 2, the annotations were generated using the Roboflow platform [12]. Polygonal annotations were preferred over simpler approaches, such as bounding boxes, because weed instances often exhibit irregular shapes, overlapping leaves, variable sizes, and complex spatial distributions. This annotation strategy provides a more precise representation of weed boundaries, making the dataset suitable for semantic segmentation, weed detection, and fine-grained analysis tasks.

Overall, the dataset captures variability across fields, seasons, UAV platforms, spatial resolutions, acquisition settings, and sensing modalities. This diversity increases the realism of the dataset and supports the evaluation of weed detection and segmentation approaches under conditions closer to real agricultural deployment.

## 4 Experimental Design, Materials and Methods

For the collection of the dataset, multiple UAV acquisition scenarios were conducted in the examined agricultural fields in Thessaloniki and Chalkidiki. The acquisition protocol was designed to include two complementary types of UAV flights: (i) orthomosaic-oriented flights, aiming at the complete coverage and reconstruction of the surveyed fields, and (ii) low-altitude image acquisition flights, aiming at the detailed image-based recording of individual plants and weed occurrences under different spatial resolutions and imaging conditions. The acquisitions were performed using two UAV platforms, namely the DJI Phantom 4 Pro equipped with an RGB camera and the DJI Mavic 3 M equipped with both RGB and multispectral (MS) cameras. The main camera characteristics and sensor specifications of the UAV platforms are summarized in Table 4.

**Table 4:** Summary of unmanned aerial vehicle and camera specifications.

Unmanned Aerial Vehicle	Characteristics
DJI Phantom 4 Pro	**RGB Camera:** 1-inch CMOS; effective pixels: 20 MP; FOV: 84 degrees; 8.8 mm / 24 mm (35 mm format equivalent); f/2.8-f/11; autofocus at 1 m-infinity.
DJI Mavic 3M	**RGB Camera:** 4/3 CMOS; effective pixels: 20 MP; FOV: 84 degrees; 24 mm equivalent focal length; aperture: f/2.8-f/11; focus: 1 m-infinity. **Multispectral Camera:** 1/2.8-inch CMOS; effective pixels: 5 MP; FOV: 73.91 degrees (61.2 degrees x 48.10 degrees); 25 mm equivalent focal length; aperture: f/2.0; fixed focus. **Bands:** Green (G): 560 ± 16 nm; Red (R): 650 ± 16 nm; Red Edge (RE): 730 ± 16 nm; Near Infrared (NIR): 860 ± 26 nm.

**Fig. 3:**
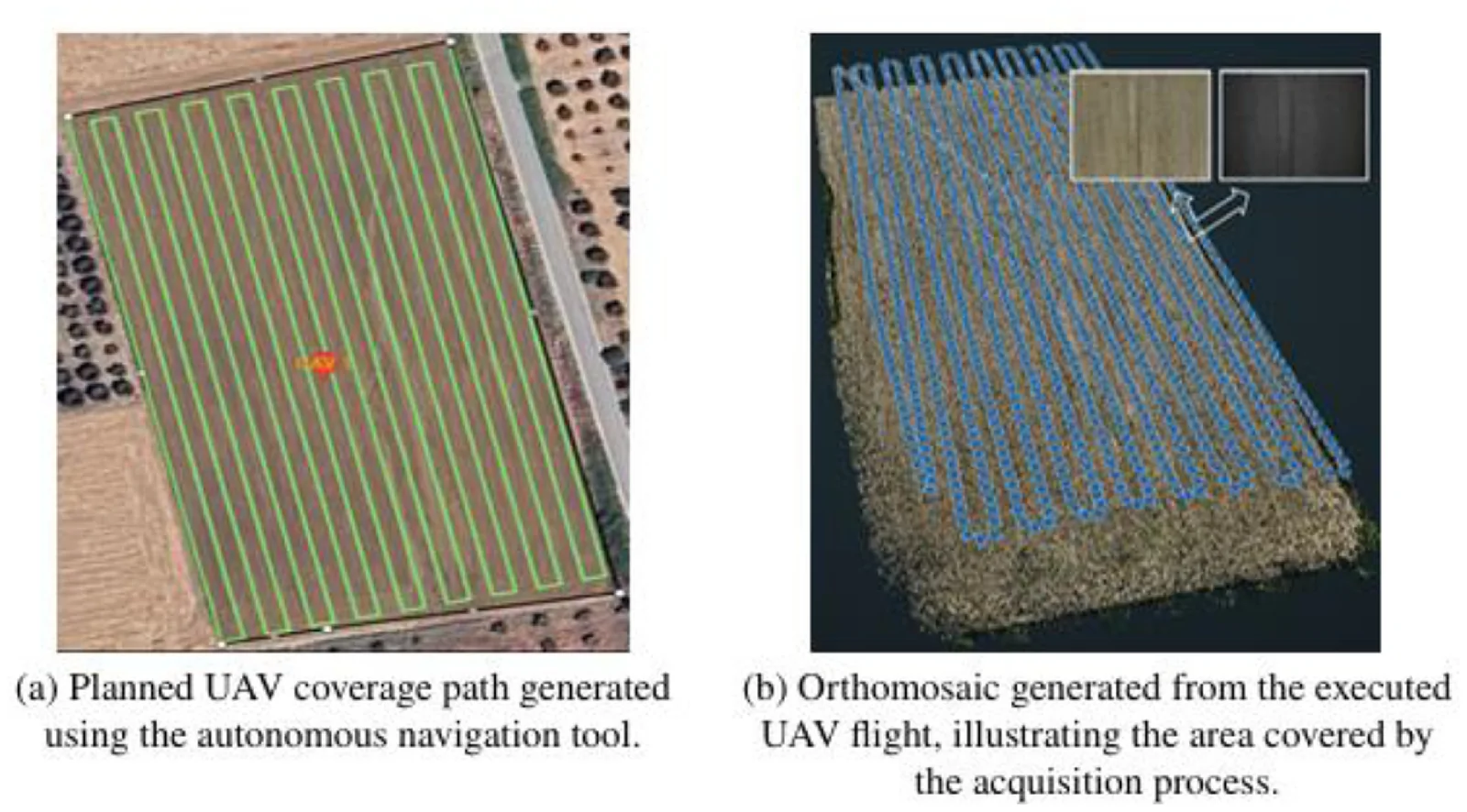
UAV-based data acquisition workflow. The first image illustrates the planned coverage path used for autonomous navigation of the DJI Mavic 3 M over the agricultural field in Chalkidiki, while the second image presents the resulting orthomosaic constructed from the captured images along the flight trajectory.

For the orthomosaic acquisitions, an autonomous UAV navigation framework was employed to ensure full coverage of the surveyed fields. The flight paths were generated using a coverage path planning approach based on a spanning tree algorithm [13], while considering UAV-specific parameters such as camera characteristics, platform dimensions, GSD, frontlap, and sidelap. These orthomosaic flights were performed in both Thessaloniki and Chalkidiki with the DJI Mavic 3 M at 20 m altitude, using both RGB and MS cameras. As reported in Table 5, the flights were planned with a GSD of 0.5 cm/pixel, 85% frontlap, and 70% sidelap. Fig. 3 illustrates both the planned coverage path followed during the UAV flight and the resulting orthomosaic generated after image stitching. The deployed UAV coverage path planning software is publicly available at https://choosepath.org/ .

**Table 5:** Summary of UAV flight parameters for the data for orthomosaic generation.

Location	Acquisition setting	Drone	Camera	GSD (cm/pixel)	Frontlap (%)	Sidelap (%)
Thessaloniki	Mavic 3 M flight at 20 m altitude RGB	Mavic 3M	RGB	0.5	85	70
	Mavic 3 M flight at 20 m altitude MS	Mavic 3M	MS	0.5	85	70
Chalkidiki	Mavic 3 M flight at 20 m altitude RGB	Mavic 3M	RGB	0.5	85	70
	Mavic 3 M flight at 20 m altitude MS	Mavic 3M	MS	0.5	85	70

In addition to the orthomosaic flights, several low-altitude UAV flights, whose acquisition parameters are summarized in Table 6, were performed to acquire detailed RGB and MS imagery of the plants at different spatial resolutions. During the summer 2025 acquisition campaign in Thessaloniki, two UAV flights were conducted at altitudes of 5 m and 10 m using the DJI Phantom 4 Pro. RGB images were acquired at a flight speed of 3 m/s, while the camera was oriented at approximately −89° with respect to the ground in order to capture a near-vertical field of view. These flights resulted in GSD values of 0.14 cm/pixel and 0.27 cm/pixel for the 5 m and 10 m flights, respectively, with 70% frontlap and 1% sidelap.

**Table 6:** Summary of UAV flight parameters for the data for weed detection.

Location	Acquisition setting	Drone	Camera	GSD (cm/pixel)
Thessaloniki	Phantom flight at 5 m altitude	Phantom 4 Pro	RGB	0.14
	Phantom flight at 10 m altitude	Phantom 4 Pro	RGB	0.27
	Phantom flight at 3 m altitude	Phantom 4 Pro	RGB	0.08
	Mavic 3 M flight 1 at 2 m altitude	Mavic 3M	RGB	0.15
	Mavic 3 M flight 1 at 2 m altitude MS	Mavic 3M	MS	0.15
	Mavic 3 M flight 2 at 2 m altitude	Mavic 3M	RGB	0.15
	Mavic 3 M flight 2 at 2 m altitude MS	Mavic 3M	MS	0.15
Chalkidiki	Phantom flight at 3 m altitude	Phantom 4 Pro	RGB	0.08
	Phantom flight at 5 m altitude	Phantom 4 Pro	RGB	0.14

During the winter 2025–2026 acquisition campaign in Chalkidiki, the same UAV platform was used to perform two additional RGB flights at altitudes of 3 m and 5 m, under similar acquisition settings. These flights resulted in GSD values of 0.08 cm/pixel and 0.14 cm/pixel. In Thessaloniki, an additional DJI Phantom 4 Pro flight was carried out at an altitude of 3 m, maintaining the same imaging characteristics and resulting in a GSD of 0.08 cm/pixel. Furthermore, two additional low-altitude flights were conducted over different parts of the Thessaloniki field using the DJI Mavic 3 M at an altitude of 2 m, with the gimbal angle adjusted to approximately −89° For each of these Mavic 3 M flights, both RGB and MS images were acquired, resulting in a GSD of 0.15 cm/pixel.

**Fig. 4:**
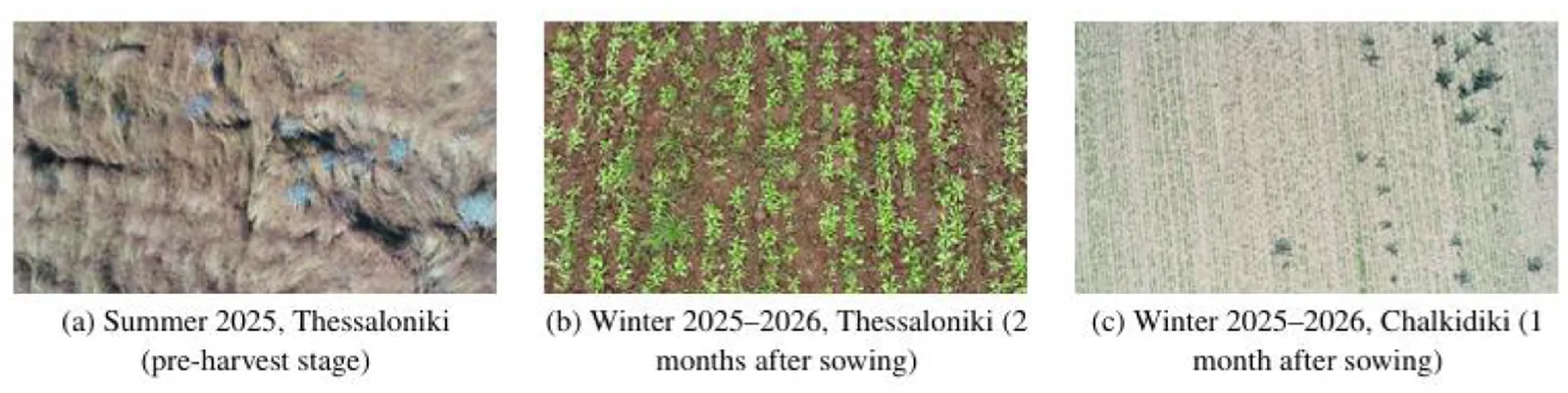
Visual diversity of the UAV image dataset, illustrating variations in season, location, crop growth stage, soil background, and weed presence in Camelina sativa cultivation.

Overall, the acquisition protocol was designed to capture weed occurrences under diverse seasonal conditions, flight altitudes, field environments, and sensing modalities. The summer acquisitions were performed at relatively higher altitudes, as the crop was closer to harvest, while the winter acquisitions were conducted at lower altitudes to better capture weed instances during the early crop growth stages, as shown in Fig. 4.

### 4.1 Dataset evaluation

The CamelinaWeed dataset [11] was evaluated using an NVIDIA GeForce RTX 5060 Ti GPU with 16 GB VRAM. To prevent data leakage, complete UAV flights were assigned exclusively to the training, validation, or geographically held-out test subset before tiling. The training subset contained 975 weed-positive and 149 weed-negative images, corresponding to approximately 15% of the positive images, and included the Thessaloniki Phantom flights conducted at 3, 5, and 10 m together with Mavic 3 M Flight 1, while Mavic 3 M Flight 2 was used for validation and the two Chalkidiki Phantom flights were reserved for testing. Weed-negative images were included to expose the models to representative background conditions and reduce false-positive detections, while their number was limited to prevent background-only samples from dominating the training process. For object detection, the bounding box associated with each polygon annotation was converted from [x,y,width,height]to normalized coordinates $\left[x_{center},y_{center},width,height\right]$using the image dimensions. RT-DETR-L [14], YOLO26m [15], YOLO26n [15], and Faster R-CNN with a ResNet-50-FPN backbone [[16], [17], [18]] were trained to detect broadleaf and narrowleaf weeds using 768 × 768 image tiles.

As shown in Table 7, RT-DETR-L achieved the highest macro performance, with a Precision of 0.862, Recall of 0.817, F1-score of 0.839, AP@50 of 0.817, and AP@50–95 of 0.717. YOLO26m achieved a comparable AP@50 of 0.803 with a processing time of 6.30 ms per tile, compared with 9.19 ms for RT-DETR-L. YOLO26n was the fastest detector at 4.20 ms per tile. Processing time represents the average time required for preprocessing and model inference on a single 768 × 768 tile using the GPU. Narrowleaf weeds achieved higher class-specific performance than broadleaf weeds across all evaluated models.

**Table 7:** Class-wise performance of the object-detection models on the geographically held-out test set.

Model	Class	Precision	Recall	F1-score	AP@50	AP@50–95	Processing time (ms/tile)
**RT-DETR-L** [14]	Broadleaf	0.846	0.813	0.829	0.792	0.710	9.20
	Narrowleaf	0.878	0.821	0.849	0.841	0.723	9.18
	**Macro average**	**0.862**	**0.817**	**0.839**	**0.817**	**0.717**	**9.19**
**YOLO26m** [15]	Broadleaf	0.809	0.701	0.751	0.771	0.630	6.31
	Narrowleaf	0.824	0.784	0.804	0.835	0.670	6.30
	**Macro average**	**0.817**	**0.743**	**0.777**	**0.803**	**0.650**	**6.30**
**YOLO26n** [15]	Broadleaf	0.731	0.649	0.688	0.721	0.550	4.20
	Narrowleaf	0.811	0.724	0.765	0.774	0.590	4.21
	**Macro average**	**0.771**	**0.687**	**0.726**	**0.748**	**0.570**	**4.20**
**Faster R-CNN ResNet-50-FPN** [[16], [17], [18]]	Broadleaf	0.782	0.669	0.721	0.730	0.510	19.00
	Narrowleaf	0.811	0.745	0.777	0.760	0.580	19.00
	**Macro average**	**0.797**	**0.707**	**0.749**	**0.745**	**0.545**	19.00

A separate instance-segmentation experiment was conducted using the same flight-wise split and the original polygon annotations. As shown in Table 8, YOLO26n-seg [15] achieved a macro mask Precision of 0.765, Recall of 0.655, F1-score of 0.706, AP@50 of 0.706, and AP@50–95 of 0.500. Overall, the evaluation demonstrates the practical usability of the dataset for training and evaluating different representative state-of-the-art weed-detection and instance-segmentation models.

**Table 8:** Class-wise mask performance of the YOLO26n-seg instance-segmentation baseline on the geographically held-out test set.

Model	Class	Mask Precision	Mask Recall	Mask F1-score	Mask AP@50	Mask AP@50–95	Processing time (ms/tile)
**YOLO26n-seg** [15]	Broadleaf	0.740	0.630	0.681	0.664	0.490	3.30
	Narrowleaf	0.790	0.680	0.731	0.748	0.510	3.30
	**Macro average**	**0.765**	**0.655**	**0.706**	**0.706**	**0.500**	**3.30**

## Limitations

The dataset was collected from three agricultural fields located in Thessaloniki and Chalkidiki, Greece, and therefore reflects the environmental and cultivation conditions of these specific regions. Weed distributions and species frequencies are naturally imbalanced across the annotated categories, resulting in unequal representation of weed classes. In addition, the dataset was acquired using specific UAV platforms, flight altitudes, and sensing configurations, which may influence the direct generalization of models trained on the dataset to substantially different acquisition conditions or agricultural environments.

## Ethics Statement

The authors have read and followed the ethical requirements for publication in Data in Brief. The current work does not involve human subjects, animal experiments, or data collected from social media platforms. UAV operations were carried out under the applicable European Union and national regulations governing unmanned aircraft flights.

## Ethics Declaration

The author(s) declare(s) that the study does not involve humans nor animal subjects.

## CRediT authorship contribution statement

**Ioannis N. Rallis:** Conceptualization, Data curation, Investigation, Software, Visualization, Writing – original draft. **Kyriakos Kempapidis:** Data curation, Formal analysis, Investigation, Validation. **Panagiotis Raptis:** Writing – review & editing, Formal analysis, Investigation, Validation. **Emmanuel K. Raptis:** Conceptualization, Data curation, Investigation, Software, Visualization, Writing – original draft, Supervision. **Athanasios Ch. Kapoutsis:** Conceptualization, Supervision, Project administration, Writing – review & editing.

## Data Availability

ZenodoCamelinaWeed: Annotated UAV Imagery Dataset for Camelina sativa (Original data).
